# Reactive-oxygen-species-mediated mechanism for photoinduced antibacterial and antiviral activities of Ag_3_PO_4_

**DOI:** 10.1186/s40543-020-00220-y

**Published:** 2020-06-11

**Authors:** Youngsik Seo, Keunchun Park, Yonggun Hong, Eun Sik Lee, Sang-Soon Kim, Yong-Tae Jung, Heonyong Park, Chian Kwon, Young-Sik Cho, Young-Duk Huh

**Affiliations:** 1 Department of Molecular Biology, Dankook University, Cheonan, 31116 Republic of Korea; 2 Department of Food Engineering, Dankook University, Cheonan, 31116 Republic of Korea; 3 Department of Microbiology, Dankook University, Cheonan, 31116 Republic of Korea; 4 Department of Chemistry, Dankook University, Cheonan, 31116 Republic of Korea

**Keywords:** Reactive oxygen species, Photoenhanced antibacterial activities, Antiviral activity, Ag_3_PO_4_

## Abstract

Cubic-shaped Ag_3_PO_4_ crystals with a mean size of 1 μm were synthesized by a precipitation method from a mixed solution of AgNO_3_, Na_2_HPO_4_, and triethanolamine. The antibacterial activities against *Escherichia coli*, *Listeria innocua*, and *Pseudomonas syringae* DC3000 in both the absence and presence of Ag_3_PO_4_ under dark conditions and in the presence of Ag_3_PO_4_ under red-light (625 nm) and blue-light (460 nm) irradiation were examined. The concentrations of reactive oxygen species (ROS) were also measured in the antibacterial action of the Ag_3_PO_4_ against *Escherichia coli.* The photoinduced enhancement of the Ag_3_PO_4_ antibacterial activity under blue-light irradiation is explained by the formation of ROS during the antibacterial action of the Ag_3_PO_4_. Moreover, the antiviral activity of Ag_3_PO_4_ against amphotropic 10A1 murine leukemia virus enhanced under blue-light irradiation via ROS production. These results provide an insight into extended bio-applications of Ag_3_PO_4_.

## Introduction

Photoenhanced catalytic and antibacterial materials have been extensively investigated in efforts to eliminate organic pollutants and microorganisms from wastewater (Chatterjee and Dasgupta 2005; Lapworth et al. 2012; Mouele et al. 2015; Schwarzenbach et al. 2006). In the process where electrons from the conduction band recombine with holes from the valence band of photocatalytic materials, reactive oxygen species (ROS) such as superoxide anions (O_2_^•−^), hydroxyl radicals (•OH), and singlet oxygen (^1^O_2_) are produced (Dickinson and Chang 2011; Li et al. 2012). These ROS play an important role in the photoenhanced catalytic activities. The ROS can also damage biomolecules and regulate cell death of microorganisms (Du and Gebicki 2004; Overmyer et al. 2003).

Silver phosphate (Ag_3_PO_4_), which has an indirect bandgap of 2.36 eV, exhibits excellent photoenhanced catalytic activity, with a quantum efficiency as high as 90% under irradiation at 420 nm (Bi et al. 2011; Chen et al. 2015). Ag_3_PO_4_ exhibits higher photocatalytic activity than TiO_2_ in the degradation of organic dyes such as methylene blue and rhodamine B (Dong et al. 2014; Liang et al. 2012). The antibacterial activity and photoinduced antibacterial activity of Ag_3_PO_4_ have also been investigated (Buckley et al. 2010; Piccirillo et al. 2015; Seo et al. 2017; Suwanprateeb et al. 2012; Wu et al. 2013). However, to the best of our knowledge, there is no report that examines antibacterial or antiviral activities arising from the ROS photoinducibly generated by Ag_3_PO_4_. In this work, we assessed the role of ROS-mediated behavior in the photoinduced antibacterial and antiviral activities of Ag_3_PO_4_ crystals against various bacteria and amphotropic 10A1 murine leukemia virus (MLV), respectively.

## Methods/experimental

### Synthesis of Ag_3_PO_4_ microcrystal

AgNO_3_ (99%, Aldrich), Na_2_HPO_4_ (99%, Aldrich), and triethanolamine (TEA; 98%, Aldrich) were used without further purification. Ag_3_PO_4_ was synthesized via a simple precipitation method at room temperature. Six milliliters of 1.0 M TEA aqueous solution was added to 30 mL of 0.01 M AgNO_3_ aqueous solution with stirring at room temperature for 10 min. Then, 20 mL of 5 mM Na_2_HPO_4_ aqueous solution was added, and the resulting mixture was stirred for 1 min at room temperature. The product was collected by centrifugation at 4000 rpm for 5 min, washed several times with water and ethanol, and then dried for 24 h at room temperature.

### Antibacterial and antiviral experimental conditions

A small fraction (10 μL) of *Escherichia coli* (*E. coli*) overnight culture was added evenly to fresh 5 mL Luria–Bertani (LB) medium containing 2 μg/mL of the Ag_3_PO_4_ product with or without 1 mM *N*-acetylcysteine (NAC, Aldrich) and then incubated in a 37 °C shaking incubator. *Pseudomonas syringae* (*P. syringae*) DC3000 was grown at 28 °C in NYGB medium (0.5% tryptone, 3% yeast extract, and 2% glycerol) containing rifampicin. Two-day grown *P. syringae* DC3000 culture was evenly aliquoted into 10 mL fresh NYGB medium containing rifampicin and further grown at 28 °C.

Antibacterial activity of Ag_3_PO_4_ to *Listeria innocua* (*L. innocua*), which was used as *L. monocytogenes* surrogate, was measured using the agar-overlay method. Bacterial culture incubated in tryptic soy broth (TSB) was inoculated to tryptic soy agar (TSA) or TSA containing Ag_3_PO_4_ (4 μg/mL). Oxford agar base (OAB; Difco, Sparks, MD) with antimicrobial supplement (Bacto Oxford antimicrobial supplement; Difco) was poured into 50-mm petri dish (bottom agar), overlaid with the inoculated TSA (top agar), and incubated at 37 °C with or without light treatment. After incubation at 37 °C for 22 h, OAB images were obtained and typical black colonies were enumerated.

For virus production, 293T human embryonic kidney cells (ATCC CRL-11268) were transiently transfected with a full-length molecular clone pMoMLV-10A1-EGFP using the CalPhos Mammalian Transfection Kit (TaKaRa Bio, Shiga, Japan). pMoMLV-10A1-EGFP is a replication-competent retroviral vector containing enhanced green fluorescent protein (EGFP). To determine the viral titer, 1 mL of virus-containing supernatants and 2 μg/mL Ag_3_PO_4_ were mixed at 37 °C for different irradiation time under the blue and red light sources. HT1080 human fibrosarcoma cells (ATCC CCL-121) were infected with 1 mL of viral supernatants at a multiplicity of infection (MOI) of 1 in the presence of 8 μg/mL polybrene. Two days after infection, green fluorescent protein (GFP)-positive cells were analyzed by a FACSCalibur^TM^ flow cytometer (Becton, Dickinson and Company, Franklin Lakes, NJ, USA).

Intracellular amounts of ROS were analyzed by fluorescence spectroscopy after reaction with 2′,7′-dichlorodihydrofluorescein diacetate (DCFH-DA). Briefly, *E. coli* cells were treated with or without Ag_3_PO_4_ under light irradiation. The cells were then additionally incubated with phosphate-buffered saline (PBS) containing 500 μM DCFH-DA for 1 h at room temperature in the dark. Finally, the amounts of ROS were measured by fluorescence spectrophotometry (Synergy HTX multimode reader; *λ*_ex_ = 485 ± 20 nm, *λ*_em_ = 528 ± 20 nm). To obtain *E. coli* images, we placed DCFH-DA-stained cells on a slide glass, covered them with a cover slip, and then observed them by fluorescence microscopy (Axioplan 2 microscope) using a green filter.

### Statistics

Data are presented as the mean ± SEM. Statistics were performed by Tukey’s post hoc test. A *p* < 0.05 is considered statistically significant.

### Instrumentation

The structure and morphology of the Ag_3_PO_4_ product were examined by powder X-ray diffraction (XRD; PANalytical X'Pert-PRO MPD) with Cu K_α_ radiation and by scanning electron microscopy (SEM; Hitachi S-4300), respectively. To examine the antibacterial activities of the Ag_3_PO_4_ product, the growth rates of *E. coli* or *P. syringae* DC3000 in the absence or presence of Ag_3_PO_4_ without light and in the presence of Ag_3_PO_4_ under blue and red light were determined by measurement of the optical density at 600 nm with a UV–vis spectrophotometer (X-ma 1200V). A blue LED (NC LED, *λ* = 460 nm) and a red LED (NC LED, *λ* = 625 nm) with equivalent luminescence were used as the blue and red light sources, respectively.

## Results and discussion

**Fig. 1:**
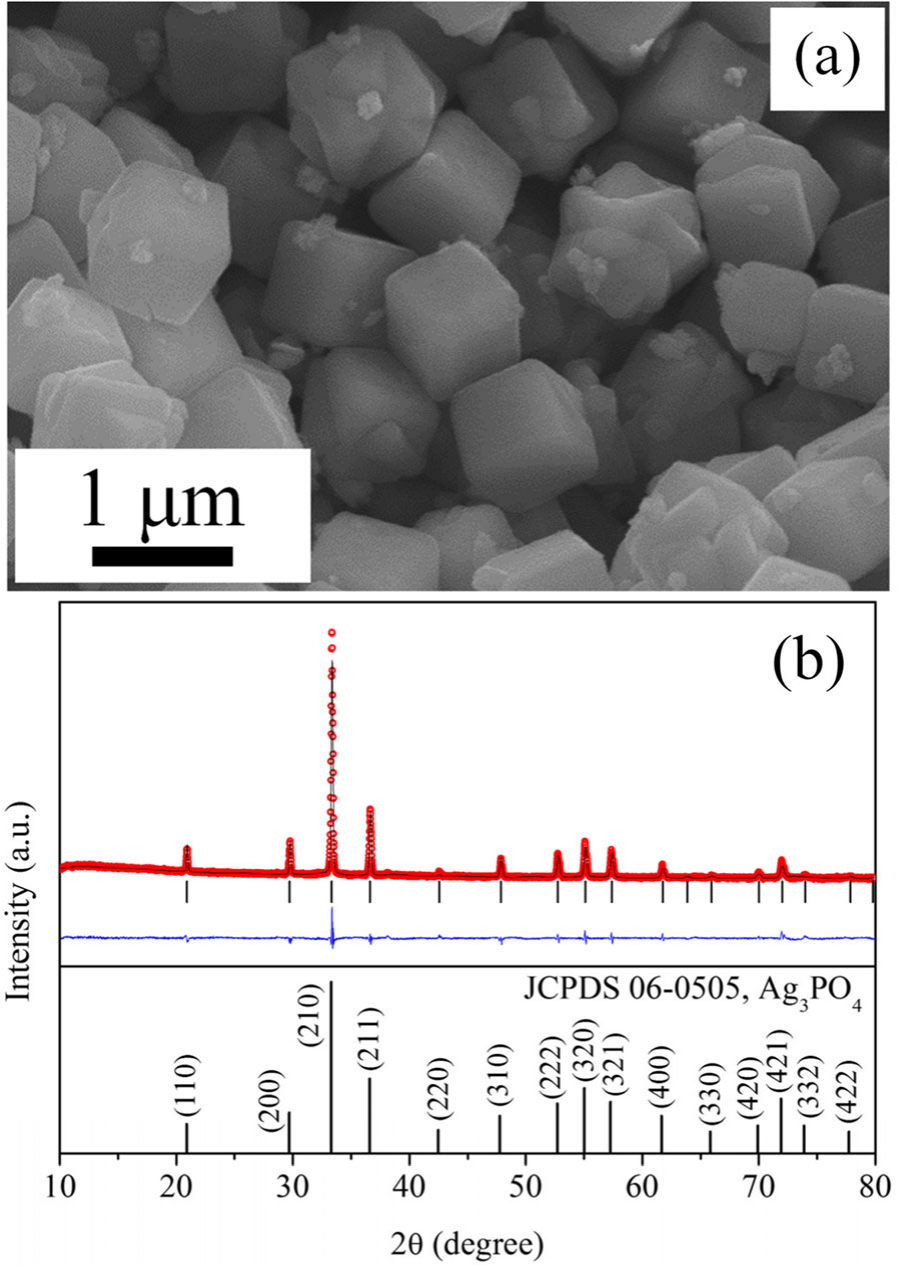
**a** SEM images of the as-synthesized Ag_3_PO_4_ product prepared by the precipitation method. **b** (Top panel) XRD patterns of the Ag_3_PO_4_ product, along with the patterns calculated using the Rietveld refinement method; the solid line (black) and open circles (red) present the measured and calculated XRD data, respectively. The intensity differences (blue) between the measured and calculated patterns are shown. The vertical markers (black) indicate the Bragg reflections. (Bottom panel) The XRD pattern and Miller indices of the cubic crystal structure of Ag_3_PO_4_ (JCPDS 06-0505) are included for comparison

Figure 1a shows an SEM image of the Ag_3_PO_4_ crystals prepared by the precipitation method at room temperature. Most of the Ag_3_PO_4_ crystals exhibit a cubic shape with a size of 1 μm. Figure 1b shows the XRD pattern of the as-synthesized Ag_3_PO_4_ crystals. The Rietveld-refined cell parameters of the Ag_3_PO_4_ crystals in this work are consistent with those of body-centered cubic Ag_3_PO_4_ with *a* = 0.6013 nm (JCPDS 06-0505).

**Fig. 2:**
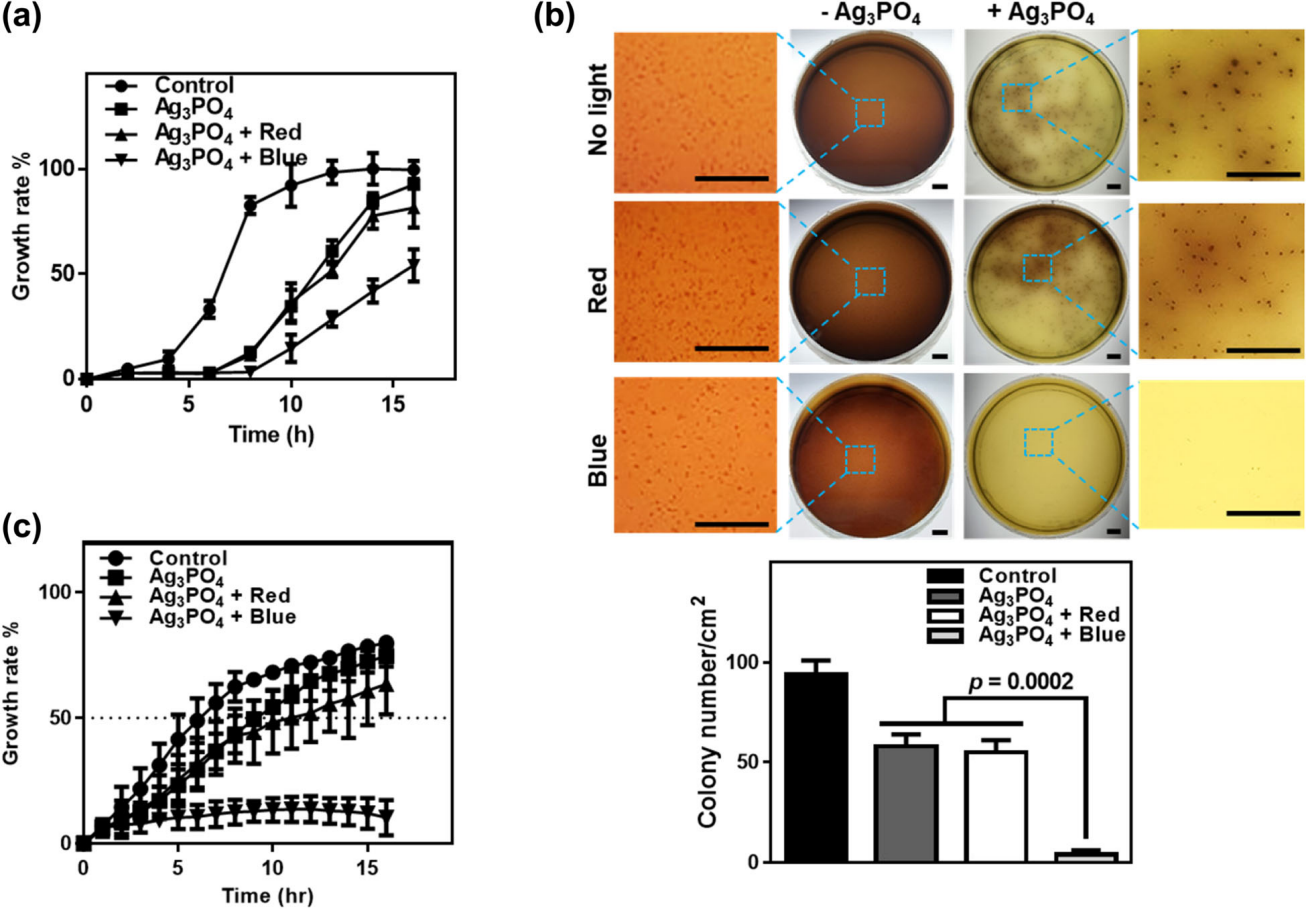
**a** The growth rate of *E. coli* in the absence (circles) or presence (squares) of Ag_3_PO_4_ under dark conditions and in the presence of Ag_3_PO_4_ under red-light (625 nm, triangles) or blue-light (460 nm, inverted triangles) irradiation. Line graphs represent mean ± SEM (*n* = 3). **b** Colony formation of *L. innocua* in the absence or presence of Ag_3_PO_4_ under dark conditions and in the presence of Ag_3_PO_4_ under red-light or blue-light irradiation. Quantified data are shown in bar graphs (mean ± SEM; *n* = 3). Scale bars are 10 μm in **b**. **c** The growth rate of *P. syringae* DC3000 in the absence (circles) or presence (squares) of Ag_3_PO_4_ under dark conditions and in the presence of Ag_3_PO_4_ under red-light (triangles) or blue-light irradiation (inverted triangles). Line graphs represent mean ± SEM (*n* = 3)

Figure 2a shows the growth rate of *E. coli* in the absence and presence of Ag_3_PO_4_ under dark conditions and in the presence of Ag_3_PO_4_ under red-light (625 nm) and blue-light (460 nm) irradiation. In the control experiment without Ag_3_PO_4_ crystals and under dark conditions, the growth rate of *E. coli* increases rapidly during the incubation period of 8 h and reaches a saturation plateau after 12–16 h. The incubation time for growth to 50% is known as the half-maximal growth time. The half-maximal growth time in the control experiment was 6.5 h for culturing the *E. coli* in the absence of Ag_3_PO_4_ and under dark conditions. When Ag_3_PO_4_ crystals were present under dark conditions, the growth rate of *E. coli* decreased compared with the growth rate in the control experiment and the half-maximal growth time increased to 11.0 h. These results indicate that the Ag_3_PO_4_ crystal exhibits antibacterial activity against *E. coli*.

In the presence of Ag_3_PO_4_ crystals and under red-light (625 nm) irradiation, an *E. coli* growth curve very similar to that for Ag_3_PO_4_ crystals under dark conditions is observed, where the half-maximal growth time is 12.0 h. Because the indirect bandgap energy of crystalline Ag_3_PO_4_ is 2.36 eV (525 nm), the red light (625 nm, 1.98 eV) lacks sufficient energy to transfer the electron from the valance band to the conduction band of the Ag_3_PO_4_ crystal. This suggests that red light does not induce photoenhancement of the antibacterial activity of Ag_3_PO_4_ crystals. However, under blue-light irradiation (460 nm, 2.70 eV), the growth rate of *E. coli* is substantially decreased in the presence of Ag_3_PO_4_ crystals and the half-maximal growth time is increased to 15.5 h. Because the blue light has sufficient energy to transfer electrons from the valance band to the conduction band of the Ag_3_PO_4_ crystals, the photoinduced enhancement of antibacterial activity of Ag_3_PO_4_ is observed only under blue-light irradiation.

Similar trends were observed for *L. innocua*, which was used as a surrogate of representative Gram-positive foodborne pathogens, *L. monocytogenes.* Figure 2b shows colonies of *L. innocua* grown on the selective agar plates in the absence and presence of Ag_3_PO_4_ under dark conditions and red-light or blue-light irradiation. Ag_3_PO_4_ decreases the number of *L. innocua* colonies by twofold under dark conditions. The colony number is about 4/cm^2^ under blue-light irradiation in the presence of Ag_3_PO_4_, when compared to 58/cm^2^ and 55/cm^2^ under dark and red-light conditions in the presence of Ag_3_PO_4_, respectively. This result indicates that blue-light irradiation remarkably and synergistically enhances the antibacterial activity of Ag_3_PO_4_. Photoinduced antibacterial activity on the agar plate gives an insight into applications of Ag_3_PO_4_ in anti-fouling and eco-friendly adhesive industry.

We then examined the photoinduced antibacterial activity of Ag_3_PO_4_ against the plant pathogenic *P. syringae* DC3000 bacterium. In Fig. 2c, the half-maximal growth rates of untreated control and Ag_3_PO_4_ under dark conditions are 6 h and 9 h, respectively. Comparatively, the half-maximal growth rates of Ag_3_PO_4_ under red-light and blue-light irradiations are 11 h and undetectable (caused by almost complete inhibition), respectively. Accordingly, Ag_3_PO_4_ under blue-light irradiation almost completely inhibits the growth of *P. syringae* DC3000, suggesting that Ag_3_PO_4_ under blue-light irradiation can be useful for crop protection from phytopathogenic bacteria.

**Fig. 3:**
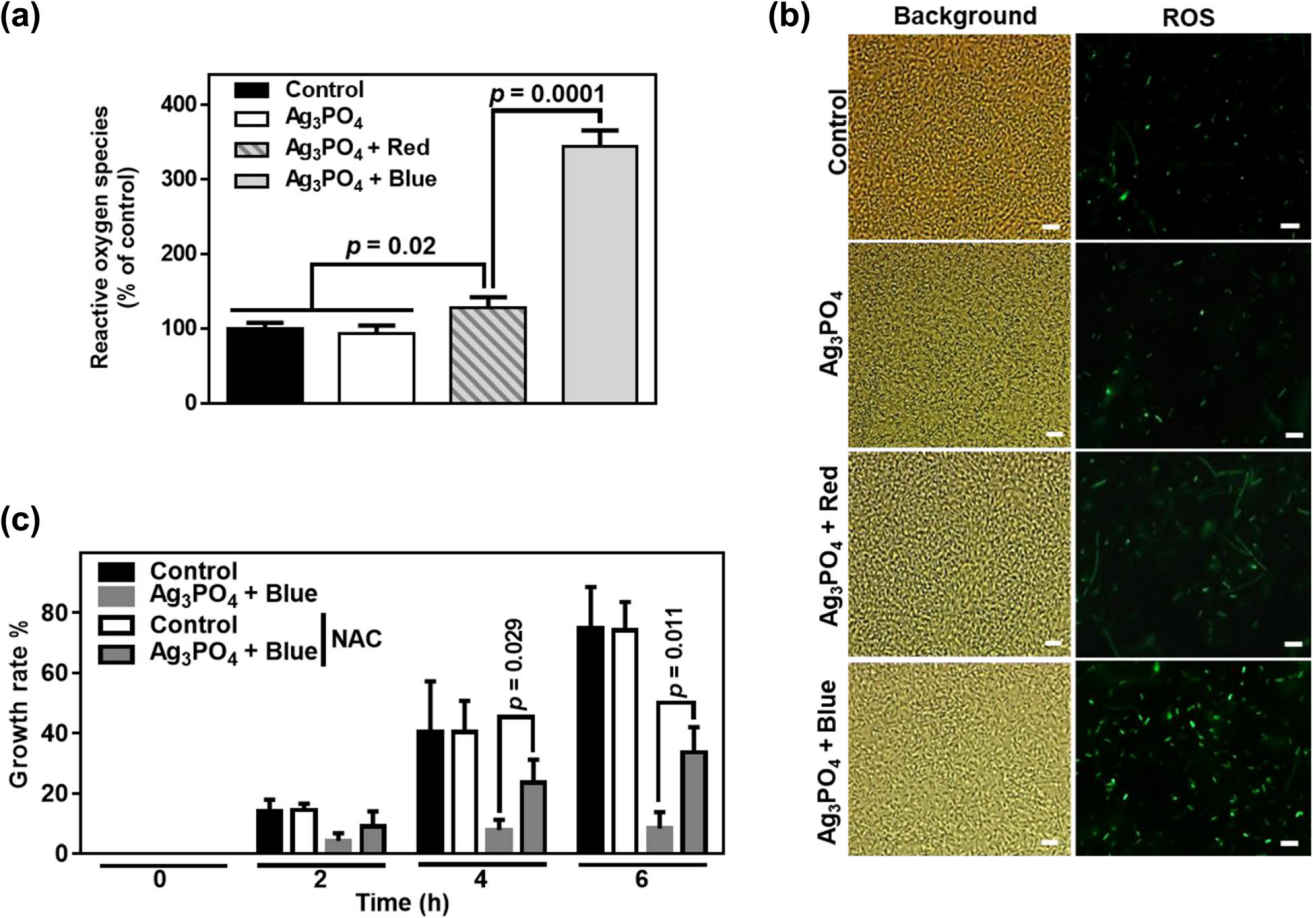
**a** The ROS concentration produced in the process of antibacterial action against *E. coli* (mean ± SEM; *n* = 3) and **b** optic (left panels showing total cells) and fluorescent (right panels) images of *E. coli* in the absence and presence of Ag_3_PO_4_ under dark conditions and in the presence of Ag_3_PO_4_ under red-light (625 nm) and blue-light (460 nm) irradiation. DCFH-DA fluorescent dye was used for ROS detection. Scale bars are 10 μm in **b**. **c** The growth rate of *E. coli* in the absence or presence of 1 mM *N*-acetylcysteine (NAC) under dark conditions and in the presence of Ag_3_PO_4_ with or without 1 mM NAC under blue-light (460 nm, inverted triangles) irradiation. Bar graphs represent mean ± SEM (*n* = 3)

To understand the mechanisms underlying the antibacterial activity of Ag_3_PO_4_ crystals, we examined whether Ag_3_PO_4_ crystals alter the levels of ROS in *E. coli*. Interestingly, Ag_3_PO_4_ crystals appeared to increase the level of ROS under blue-light irradiation, whereas Ag_3_PO_4_ crystals alone or under red light exhibited no effect, as shown in Fig. 3. Quantified amounts of ROS and ROS-stained *E. coli* cells are shown in Fig. 3a and b, respectively. In both panels, the level of ROS was highest in *E. coli* cells exposed to Ag_3_PO_4_ crystals in conjunction with blue-light irradiation. These data indicate that the antibacterial activity of Ag_3_PO_4_ under blue-light irradiation corresponds to the amount of ROS in *E. coli*. More convincingly, *N*-acetylcysteine (NAC) known as an ROS scavenger reverses the antibacterial activity of Ag_3_PO_4_ under blue-light irradiation as shown in Fig. 3c.

**Fig. 4:**
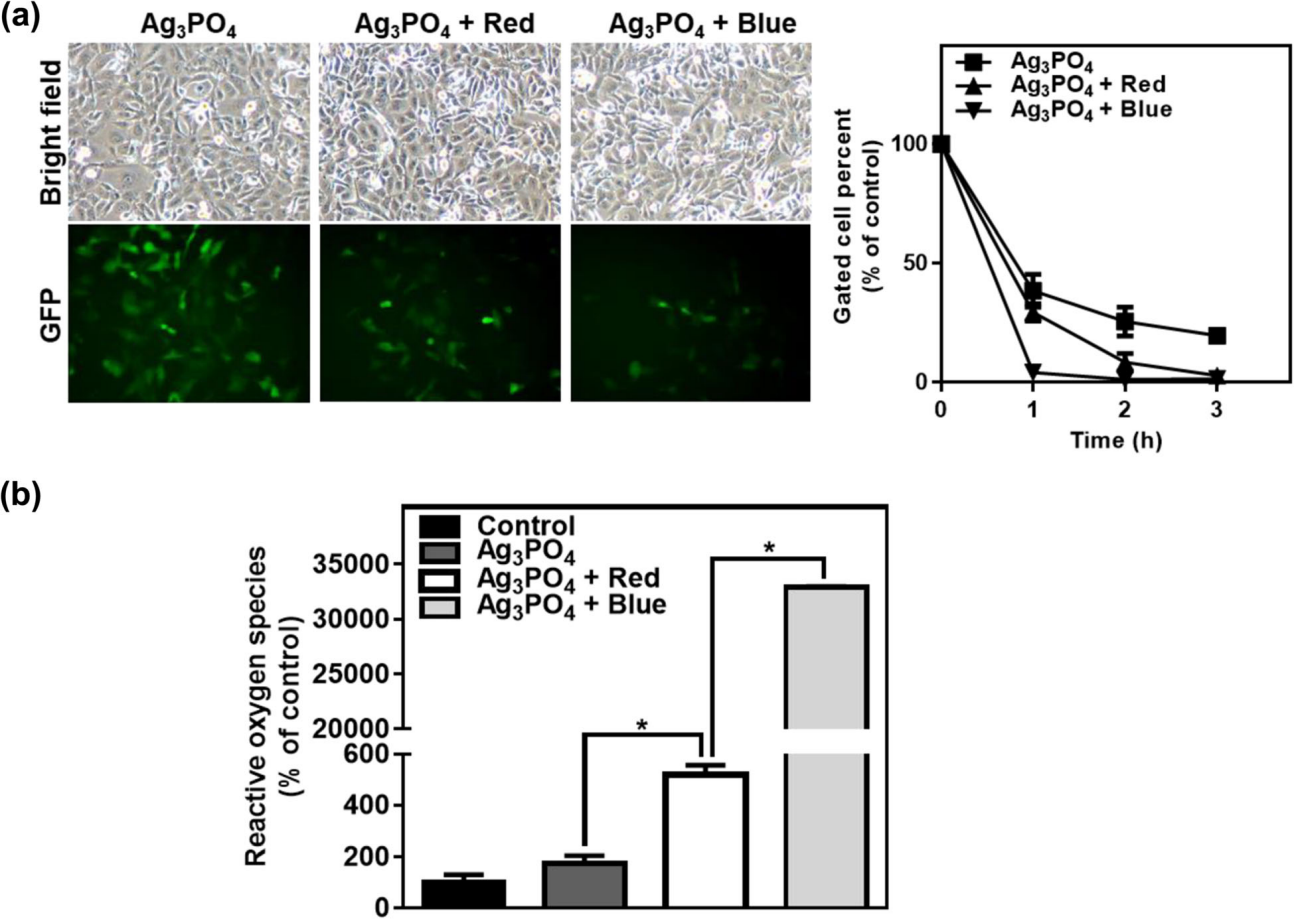
**a** Antiviral activity of Ag_3_PO_4_ under red-light (625 nm) and blue-light (460 nm) irradiation. HT1080 cells were inoculated with MoMLV-10A1-EGFP at an MOI of 1. Viral supernatants were collected after mixing with Ag_3_PO_4_ under no light, red-light, and blue-light irradiation. Representative cells observed under optic and fluorescence microscopy are shown in the left panel. Forty-eight hours after infection, the GFP-expressed cells were analyzed by flow cytometry. Fluorescence-gated cell percents are shown in line graphs in the right panel (mean ± SEM; *n* = 3). **b** The ROS concentrations generated by Ag_3_PO_4_ suspended in PBS under dark conditions and under red-light or blue-light irradiation. Quantified data are shown in bar graphs (mean ± SEM; *n* = 3) *p < 0.0001

We furthermore examined the antiviral activity of Ag_3_PO_4_ under blue-light irradiation. Figure 4a shows that amphotropic 10A1 murine leukemia virus (MLV) was more severely inactivated by Ag_3_PO_4_ under blue-light irradiation, when compared to Ag_3_PO_4_ under dark conditions and Ag_3_PO_4_ under red-light irradiation. We assume that inactivation of the MLV by blue-light irradiated Ag_3_PO_4_ might be attributable to the peroxidation of the envelope membrane phospholipids, which is furthermore detrimental to DNA (Paiva and Bozza 2014). Given that the envelop membrane phospholids are damaged by blue-light irradiated Ag_3_PO_4_, other enveloped viruses including HIV-1, SARS-CoV, MERS-CoV, and SARS-CoV2 can be inactivated by blue-light irradiated Ag_3_PO_4_. To understand the antiviral activity of Ag_3_PO_4_ under blue-light irradiation, the possibility of the generation of ROS was examined when blue light irradiates on the Ag_3_PO_4_ solution. Figure 4b shows that ROS is substantially increased by photoinduction to the Ag_3_PO_4_ solution. This result supports that the ROS is detrimental to viral particles.

## Conclusion

We synthesized cubic Ag_3_PO_4_ crystals with a mean size of 1 μm to investigate their antibacterial and antiviral activities. The Ag_3_PO_4_ crystals showed good antibacterial and antiviral activities against *E. coli*, *L. innocua*, *P. syringae* DC3000, and amphotropic 10A1 MLV. The photoinduced enhancement of the antibacterial and antiviral activities of Ag_3_PO_4_ under blue-light irradiation was observed. The ROS mediation process in the antibacterial and antiviral activities was confirmed through measurements of the concentrations of ROS. The formation of ROS plays an important role in the antibacterial and antiviral activities of Ag_3_PO_4_. These findings suggest that the photoinduced enhancement of antibacterial and antiviral activities of Ag_3_PO_4_ can be used for the biomedical application including anti-fouling, additives, and crop cultivations.

## Data Availability

Not applicable

## Abbreviations

DCFH-DA: 2′,7′-Dichlorodihydrofluorescein diacetate
*E. coli*: *Escherichia coli*
EGFP: Enhanced green fluorescent protein
GFP: Green fluorescent protein
*L. innocua*: *Listeria innocua*
LB: Luria–Bertani
MLV: Murine leukemia virus
MOI: Multiplicity of infection
NAC: *N*-Acetylcysteine
OAB: Oxford agar base
PBS: Phosphate-buffered saline
*P. syringae*: *Pseudomonas syringae*
ROS: Reactive oxygen species
SEM: Scanning electron microscopy
TEA: Triethanolamine
TSA: Tryptic soy agar
TSB: Tryptic soy broth
XRD: X-ray diffraction

## References

[CR1] Bi Y, Ouyang S, Cao J, Ye J (2011). Facile synthesis of rhombic dodecahedral AgX/Ag_3_PO_4_ (X = Cl, Br, I) heterocrystals with enhanced photocatalytic properties and stabilities. Phys Chem Chem Phys..

[CR2] Buckley JJ, Lee AF, Olivic L, Wilson K (2010). Hydroxyapatite supported antibacterial Ag_3_PO_4_ nanoparticles. J Mater Chem..

[CR3] Chatterjee D, Dasgupta S (2005). Visible light induced photocatalytic degradation of organic pollutants. J Photochem. Photobiol C: Photochem Rev..

[CR4] Chen X, Dai Y, Wang X (2015). Methods and mechanism for improvement of photocatalytic activity and stability of Ag_3_PO_4_: a review. J Alloys Compd..

[CR5] Dickinson BC, Chang CJ (2011). Chemistry and biology of reactive oxygen species in signaling or stress responses. Nat Chem Biol..

[CR6] Dong L, Wang P, Wang S, Lei P, Wang Y (2014). A simple way for Ag_3_PO_4_ tetrahedron and tetrapod microcrystals with high visible-light-responsive activity. Mater Lett..

[CR7] Du J, Gebicki JM (2004). Proteins are major initial cell targets of hydroxyl free radicals. Int J Biochem Cell Biol..

[CR8] Lapworth DJ, Baran N, Stuart ME, Ward RS (2012). Emerging organic contaminants in groundwater: a review of sources, fate and occurrence. Environ Pollut..

[CR9] Li Y, Zhang W, Niu J, Chen Y (2012). Mechanism of photogenerated reactive oxygen species and correlation with the antibacterial properties of engineered metal-oxide nanoparticles. ACS Nano..

[CR10] Liang Q, Ma W, Shi Y, Li Z, Yang X (2012). Hierarchical Ag_3_PO_4_ porous microcubes with enhanced photocatalytic properties synthesized with the assistance of trisodium citrate. CrystEngComm..

[CR11] Mouele ESM, Tijani JO, Fatoba OO, Petrik LF (2015). Degradation of organic pollutants and microorganisms from wastewater using different dielectric barrier discharge configurations—a critical review. Environ Sci Pollut Res..

[CR12] Overmyer K, Brosché M, Kangasjärvi J (2003). Reactive oxygen species and hormonal control of cell death. Trends Plant Sci..

[CR13] Paiva CN, Bozza MT (2014). Are reactive oxygen species always detrimental to pathogens?. Antiox Redox Signal..

[CR14] Piccirillo C, Pinto RA, Tobaldi DM, Pullar RC, Labrincha JA, Pintado MME, Castro PML (2015). Light induced antibacterial activity and photocatalytic properties of Ag/Ag_3_PO_4_-based material of marine origin. J Photochem Photobiol A: Chem..

[CR15] Schwarzenbach RP, Escher BI, Fenner K, Hofstetter TB, Johnson CA, Gunten UV, Wehrli B (2006). The challenge of micropollutants in aquatic systems. Science..

[CR16] Seo Y, Yeo BE, Cho YS, Park H, Kwon C, Huh YD (2017). Photo-enhanced antibacterial activity of Ag_3_PO_4_. Mater Lett..

[CR17] Suwanprateeb J, Thammarakcharoen F, Wasoontararat K, Chokevivat W, Phanphiriya P (2012). Preparation and characterization of nanosized silver phosphate loaded hydroxyapatite by single step co-conversion process. Mater Sci Eng C..

[CR18] Wu A, Tian C, Chang W, Hong Y, Zhang Q, Qu Y, Fu H (2013). Morphology-controlled synthesis of Ag_3_PO_4_ nano/microcrystals and their antibacterial properties. Mater Res Bull..

